# A hypoxia-related prognostic model predicts overall survival and treatment response in hepatocellular carcinoma

**DOI:** 10.1042/BSR20221089

**Published:** 2022-11-18

**Authors:** Jiyuan Xing, Shen Shen, Xiaorui Liu, Yang Zhang

**Affiliations:** 1 Gene Hospital of Henan Province, Precision Medicine Center, The First Affiliated Hospital of Zhengzhou University, Zhengzhou, China; 2 Department of Infectious Diseases, The First Affiliated Hospital of Zhengzhou University, Zhengzhou, China; 3 Department of Geriatric Respiratory and Sleep, The First Affiliated Hospital of Zhengzhou University, Zhengzhou 450052, China

**Keywords:** HCC, hypoxia, hypoxia-related genes, prognostic model, therapeutic response

## Abstract

Hypoxia and hypoxia-related genes regulate tumor initiation and progression. However, the exact roles that hypoxia plays in hepatocellular carcinoma (HCC) remain unclear. In the present study, we calculated the hypoxia score of each sample in the GSE14520 training set by single-sample gene set enrichment analysis (ssGSEA). Then, weighted gene coexpression network analysis (WGCNA) was utilized to identify gene modules most correlated with hypoxia. Least absolute shrinkage and selection operator (LASSO) Cox regression analysis was utilized to further compress the candidate genes. We constructed the hypoxia-related prognostic risk score (HPRS) model based on the genes’ corresponding Cox regression coefficients. Univariate and multivariate Cox analyses of the hypoxia score and clinicopathological characteristics showed that the hypoxia score and stage were the main risk factors affecting the overall survival of patients. Based on WGCNA, we identified 41 key hypoxia-related gene modules and screened out nine core genes to construct the HPRS model. Importantly, high-HPRS patients have a worse prognosis, while low-HPRS patients have a better prognosis. Further research showed that various immune cells, such as CD8 T cells, cytotoxic cells, and DCs, were significantly enriched in the low-HPRS group compared with the high-HPRS group. Notably, patients in the low-HPRS group were less likely to benefit from immunotherapy and chemotherapy than those in the high-HPRS group. In summary, we identified and validated a hypoxia-derived gene model that could serve as a potential biomarker to predict prognosis and therapeutic response in HCC.

## Introduction

Hepatocellular carcinoma (HCC) is one of the most widespread cancers and ranked as the fourth leading cause of cancer-related death in 2018 [1]. Approximately 80–90% of neoplasms develop in patients with cirrhosis; thus, therapeutic options might be limited because of the poor health status of patients [2]. Despite substantial improvements in the diagnosis, prevention, and management of HCC over the past decade, the prognosis of HCC remains poor. The 5-year overall survival rate for HCC patients is 20% [3]. Therefore, it is imperative to deeply understand the molecular mechanisms, effectively assess the prognostic risk, and identify therapeutic targets that would greatly benefit HCC treatment strategies.

Hypoxia, a condition of insufficient oxygen, is a typical hallmark of the microenvironment of nearly all solid tumors [4]. The presence of hypoxic regions is an important prognostic factor for human cancers. Accumulating studies have confirmed that hypoxia is not only related to the plasticity and heterogeneity of tumors but also induces a more aggressive and metastatic phenotype [5]. Severe hypoxia has been detected in the HCC context. HCC usually occurs in cirrhosis induced by chronic liver injury and increases fibrinogenesis production, leading to decreased vascularization and hypoxia [6]. Furthermore, hypoxia is considered to be involved in HCC development (such as tumor size, tumor-node-metastasis (TNM) stage, and lymph node metastasis) and therapy (including radiotherapy, chemotherapy, and immunotherapy) [7,8]. However, the exact roles that hypoxia plays in HCC remain unclear. The main reason is that it is difficult to detect pO_2_ in patients with liver cancer. In addition, there is no specific receptor or signaling pathway for hypoxia, and the mechanism of hypoxia is extremely complicated [9]. Recently, with advancements in sequencing technology, research on molecular mechanisms based on bioinformatics analysis has become one of the key methods for cancer research [10,11]. Some groups have constructed prognostic models based on complex hypoxia molecules in HCC [12,13]. Therefore, it is possible and meaningful to find potential biomarkers for the diagnosis and survival prediction of HCC to improve patient stratification and optimize treatment strategies.

Here, we collected hypoxia-related genes in HCC from The Cancer Genome Atlas (TCGA), GSE14520, GSE76427, and International Cancer Genome Consortium (ICGC) datasets. Then, single-sample gene set enrichment analysis (ssGSEA) was used to quantify the hypoxia feature scores of patients with HCC in the training dataset GSE14520. Based on weighted gene coexpression network analysis (WGCNA), we identified 41 key hypoxia-related gene modules and then screened out the core genes according to the correlation analysis between gene expression in the pink module and the hypoxia score. Finally, a total of nine prognostic hypoxia-related genes were identified and used to construct the hypoxia-related prognostic risk score (HPRS) model, which was an independent risk factor. Furthermore, HPRS was found to exert stable predictive performance for immunotherapy and chemotherapy of HCC.

## Methods

### Data collection

A total of 365 HCC patients with clinical information and mRNA expression patterns were obtained from the TCGA-LIHC dataset and the GSE14520 (*n*=221) and GSE76427 datasets (*n*=115), which were retrieved from the Gene Expression Omnibus (GEO) database. In addition, we downloaded the ICGC-LIRI-JP dataset from the HCCDB database, which included 212 HCC samples. In the present study, we used GSE14520 as the training dataset and TCGA, ICGC, and GSE76427 as the independent validation datasets. Hypoxia-related genes were obtained from the Molecular Signatures Database (MSigDB) glycolysis pathway ‘Hallmark-hypoxia.’

### Data preprocessing

The RNA sequencing (RNA-seq) data of TCGA-LIHC were preprocessed in the following steps:

(i) Samples without survival time were excluded; (ii) samples without living status were excluded; (iii) ‘Ensembl’ was converted into ‘Gene symbol’; and (iv) the median value for the expression of multiple Gene Symbols was used.

We downloaded the annotation information of the corresponding chip platform, mapped the probes to the genes according to the annotation information, and removed the probes that matched one probe to multiple genes. When multiple probes matched a gene, the median was used as the gene expression value.

### Cell culture and quantitative reverse-transcription PCR

To explore the expression level of the key genes in HCC cell line (Hep G2) and normal human liver cell line LO2, we used the method quantitative reverse-transcription PCR (qRT-PCR). The detailed processes have been described in our previous article. The primers are listed in Supplementary Table S1.

### WGCNA

WGCNA is a method for exploring the gene expression patterns of a large number of samples that can group genes with similar expression patterns and analyze the association between modules and specific characteristics or phenotypes. Therefore, it is mainly used to explore the association between disease characteristics and gene expression levels [14,15]. In the present study, WGCNA was utilized to identify the gene module that was most correlated with hypoxia based on the correlation between the module feature vector and the hypoxia score.

### Least absolute shrinkage and selection operator

Least absolute shrinkage and selection operator (LASSO) is a compression estimation method [16]. It is used to construct more refined models by compressing some coefficients via a penalty function. It is mainly used to select variables in parameter estimation and can better solve the multicollinearity problem in regression analysis [17]. We used the R software package ‘glmnet’ to perform 1000 LASSO Cox regression analyses to compress the candidate genes to reduce the number of genes in the risk model.

### Gene set enrichment analysis

Gene set enrichment analysis (GSEA) was used to judge the influence of the candidate genes on phenotypic changes by evaluating the distribution trends of the candidate genes in the gene table ranked by phenotypic correlation [18,19]. GSEA software was downloaded from http://software.broadinstitute.org/gsea/index.jsp. In the present study, we analyzed the different activation pathways in the high- and low-HPRS groups. We performed GSEA using all candidate gene sets in the Hallmark database [20,21]. We defined false discovery rate (FDR) <0.05 as significant enrichment.

### Calculation of the infiltrating abundance of tumor microenvironment cells

ssGSEA was utilized to calculate the degree of enrichment of the gene set in each sample within a given dataset [22]. We performed ssGSEA to calculate the abundance of immune cell infiltration in the tumor microenvironment (TME) [23,24]. Here, the immune cell gene set used to mark each infiltrating immune cell type was from Charoentong [25]. In addition, ESTIMATE software was used to quantify the relative abundance of immune cells in HCC.

### Construction of the HPRS model

(1) First, we assessed the hypoxia score of each sample in the GSE14520 dataset. The ‘GSVA’ R package was used for ssGSEA. The gene set ‘Hallmark-hypoxia’ downloaded from the MSigDB was used for GSVA analysis. (2) The gene expression profile of the GSE14520 dataset was used. WGCNA was employed to explore the correlation between the feature vector of the module and the hypoxia score in the GSE14520 dataset. We identified the most relevant module for hypoxia as the hypoxia-related module. (3) Next, we sought to identify hypoxia-related genes also related to prognosis. We identified the core genes in the module. According to the relationship between the core genes and the prognosis of patients, we included the most significant prognostic genes in the prognostic hypoxia-related gene signature. (4) Finally, we constructed the HPRS model. After obtaining the prognostic value of each gene feature score, we used the following formula to calculate the HPRS score of each patient: HPRS =(β×Expi), where i refers to the gene expression level of the prognostic hypoxia-related gene, and β is the gene’s corresponding Cox regression coefficient.

### Statistical analysis

GraphPad Prism 8.0 (San Diego, CA, USA) and R software (version 3.6.1) were utilized for statistical analysis and plotting graphs. Continuous variables are summarized as the mean ± standard deviation. Student’s *t* test or one-way analysis of variance was used to assess differences between groups. Kaplan–Meier analysis was used to draw survival curves. Univariate Cox regression was applied to calculate the proportional hazards of the factors to overall survival. Multivariable Cox regression was used to identify the independent prognostic risk factors. Patients with thorough relevant data were qualified for further analysis with a multivariate model.

## Results

### Hypoxia is the main risk factor for the prognosis of patients with HCC

**Figure 1:**
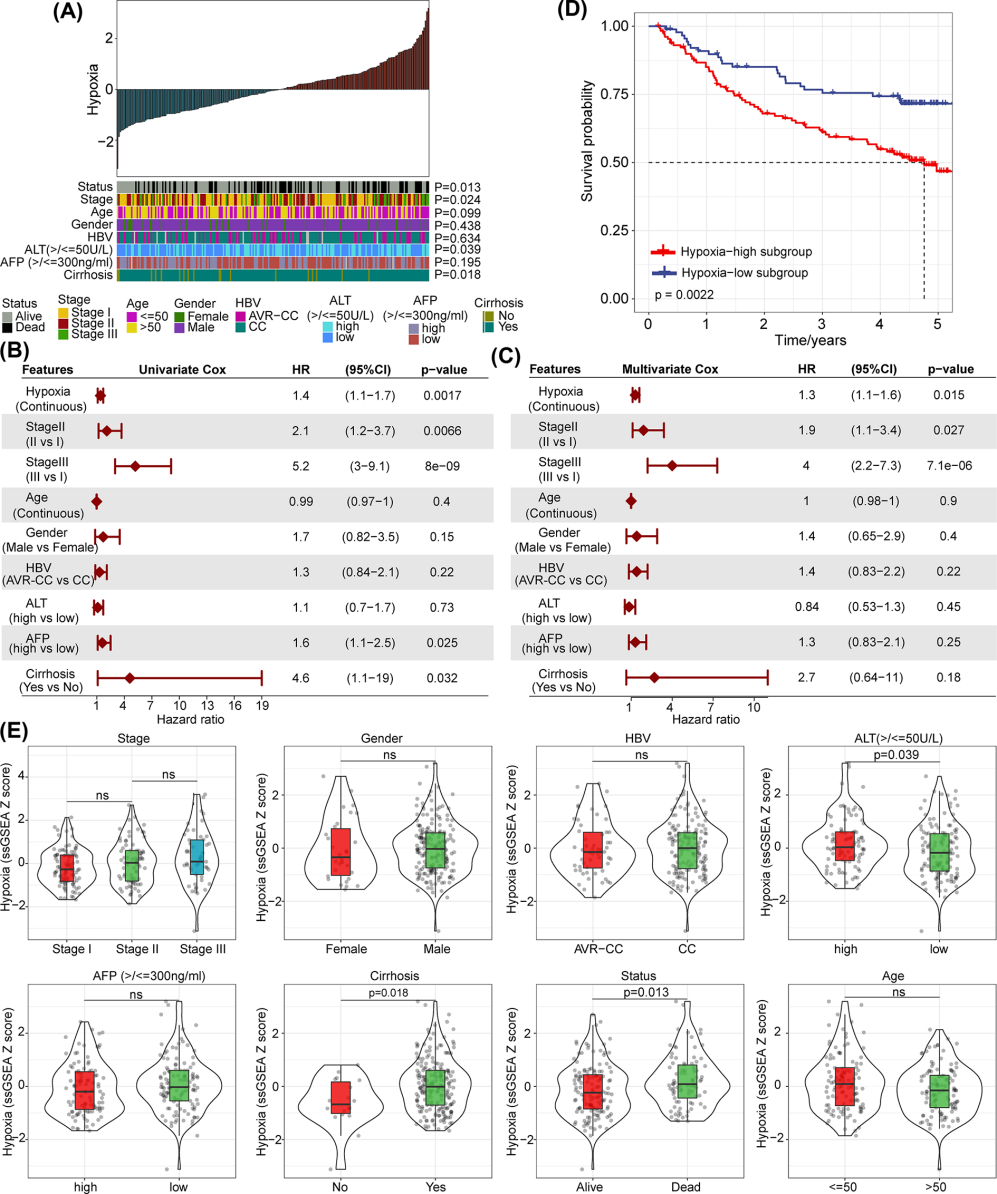
Hypoxia is a dominant risk factor for the prognosis of patients with HCC (**A**) Correlation analysis between the hypoxia score and clinicopathological characteristics of the GSE14520 cohort. (**B,C**) Univariate and multivariate Cox analyses showed that hypoxia was an independent risk factor for overall survival among clinicopathological characteristics. (**D**) The difference in the overall survival rate of patients in different scoring groups. (**E**) The hypoxia scores between different stage groups, ALT groups, cirrhosis groups, and living status groups were significantly different.

To explore the effect of hypoxia on the prognosis of patients with HCC, we identified a hypoxia gene set based on the Hallmark-hypoxia gene set in MSigDB. Then, ssGSEA was used to quantify the hypoxia feature scores of HCC patients in the GSE14520 training dataset. The results of the correlation between the hypoxia score and clinicopathological characteristics of HCC patients showed that the hypoxia score was significantly associated with stage, alanine aminotransferase (ALT), cirrhosis, and the overall survival status of patients (Figure 1A). Univariate and multivariate Cox analyses of the hypoxia score and clinicopathological characteristics showed that the hypoxia score and stage were the main risk factors affecting the overall survival rate of patients (Figure 1B,C). Next, we divided the HCC samples in GSE14520 into high- and low-score groups based on the hypoxia score from ssGSEA. The high-score group had poorer overall survival than the low-score group (*P*=0.0022) (Figure 1D). In addition, we compared whether there were differences in the hypoxia scores between different clinicopathological groups. We found that the hypoxia scores of patients in stage III had not significant difference than those of patients in stage I or stage II (Figure 1E). The hypoxia scores of the ALT, cirrhosis, and living status groups were also significantly different (Figure 1E). Overall, hypoxia was found to be a key risk factor for the overall survival of HCC patients, and patients with a high hypoxia score tend to have a poor prognosis.

### Identification of key hypoxia-related gene modules

**Figure 2:**
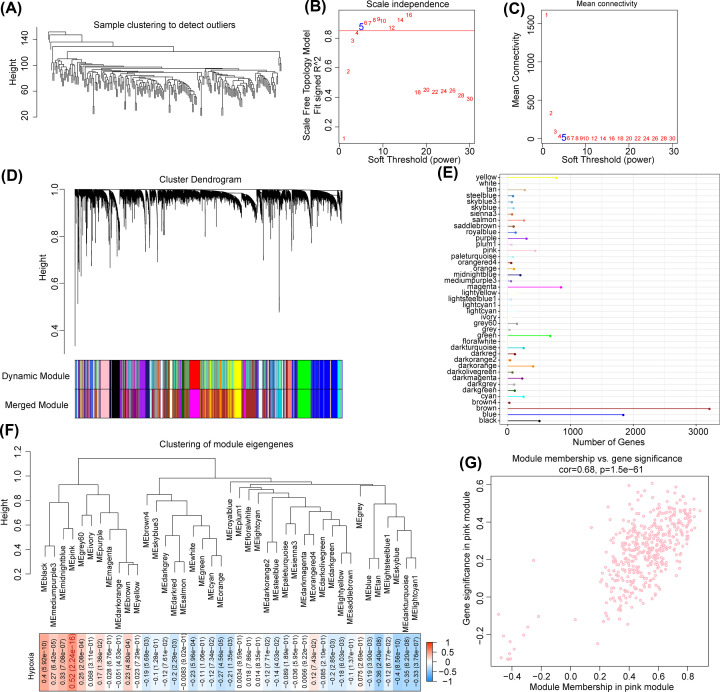
Identification of key hypoxia-related gene modules (**A**) Samples from the GSE14520 cohort were clustered using the R software package ‘WGCNA.’ (**B,C**) The coexpression network is a scale-free network when β = 5. (**D**) A total of 41 modules were obtained by the WGCNA method. (**E**) The gene statistics of each module. (**F**) Of the 41 modules, there was a significant positive correlation between the pink module and hypoxia by correlation analysis. (**G**) Module membership and gene significance in the pink module were markedly positively correlated.

To identify key hypoxia-related gene modules, we clustered the samples from GSE14520 using the R software package ‘WGCNA’ (Figure 2A). To ensure that the coexpression network is a scale-free network and that the correlation coefficient is greater than 0.85, we chose β = 5 (Figure 2B,C). Next, we clustered genes based on the standard hybrid dynamic shearing tree. Then, we calculated the eigengenes of each module. Cluster analysis was performed on the modules, merging the modules that were closer to each other into a new module and setting height = 0.3, DeepSplit = 2, and minModuleSize = 30. A total of 41 modules were obtained (Figure 2D). The gene statistics of each module are shown in Figure 2E. Furthermore, we analyzed the correlation between each module and the hypoxia score. We found that of the 41 modules, the pink module was significantly positively associated with hypoxia (*r* = 0.52, *P*=2.24 × 10^-16^) (Figure 2F). Therefore, we defined the pink module as a hypoxia-related gene module. Module membership (MM) was markedly positively correlated with gene significance (GS) (*r* = 0.68, *P*<1 × 10^-5^) (Figure 2G). In summary, we identified the pink module as the key gene module for hypoxia.

### Screening prognostic hypoxia-related genes

**Figure 3:**
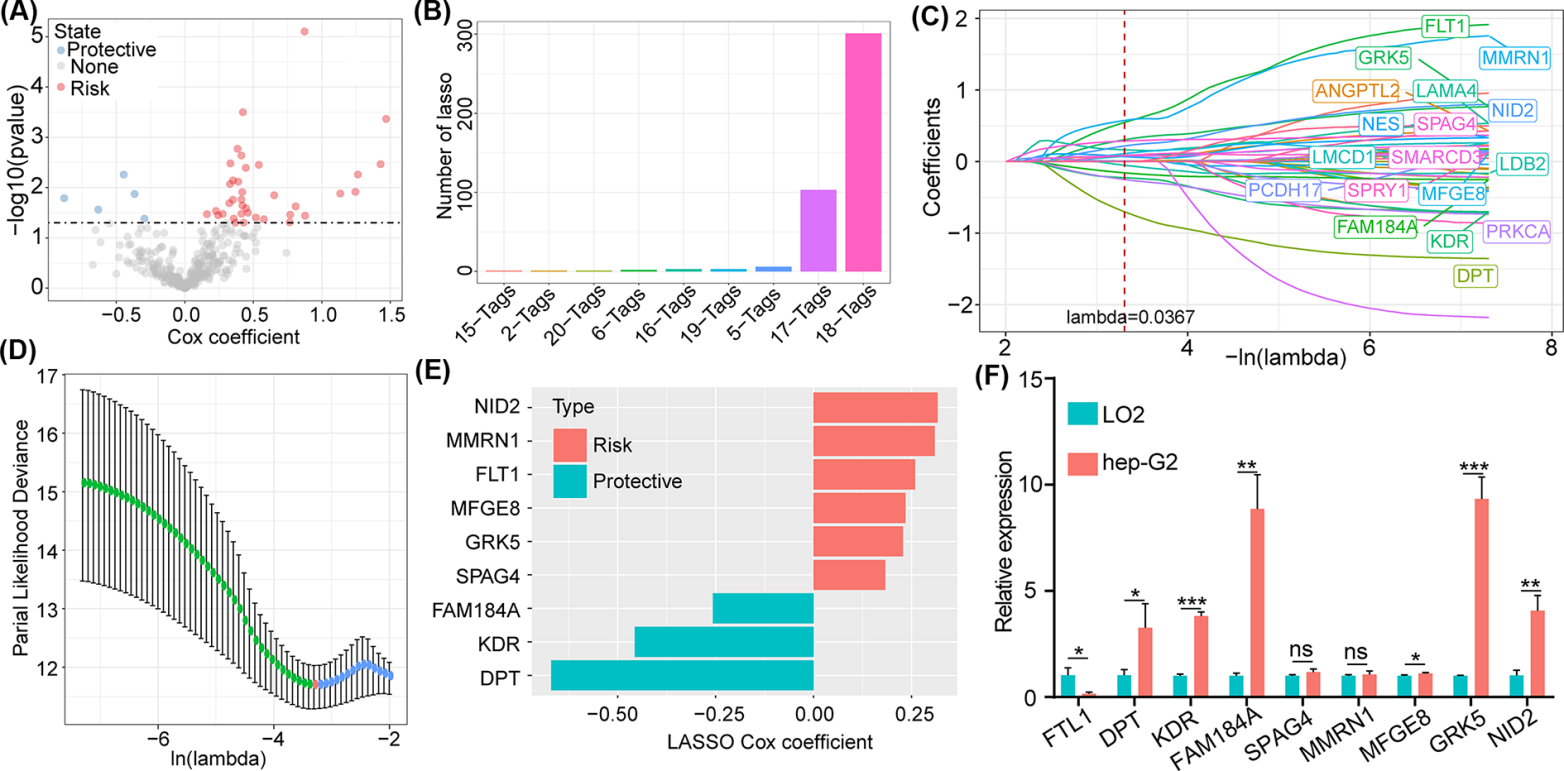
Screening for prognostic hypoxia-related genes (**A**) We identified 42 genes that were associated with prognosis (*P*<0.05), including 37 risk genes and five protective genes, by using univariate regression analysis. (**B**) LASSO regression to further compress the 42 genes in the GSE14520 training set. (**C**) The value of each independent variable. (**D**) The confidence interval (CI) under each lambda is analyzed. (**E**) A total of nine genes were defined as prognostic hypoxia-related genes through multivariate regression analysis. (**F**) The expression of five core genes in HepG2 cell line compared and normal cell line LO2.

After identifying the key hypoxia-related gene module, we screened out the core genes according to the correlation analysis between gene expression in the pink module and the hypoxia score. Afterward, we used univariate regression analysis and identified 42 genes that were associated with prognosis (*P*<0.05), including 37 risk genes and five protective genes (Figure 3A). Then, we used LASSO regression to further compress the 42 genes in the GSE14520 database. We observed that the combination of 18 genes appeared the most frequently (Figure 3B), and we further analyzed the value of each independent variable (Figure 3C). We used ten-fold cross-validation to overcome overfitting. The model reached optimum performance when lambda was 0.0367 and the number of genes was 18 (Figure 3D). In addition, we identified nine genes (DPT, FAM184A, KDR, FLT1, GRK5, MFGE8, MMRN1, NID2, and SPAG4) as prognostic hypoxia-related genes through multivariate regression analysis of the 18 genes (Figure 3E). Finally, we found the NID2, GRK5 MFGE8, KDR, DPT, and FM184A were highly expressed in HepG2 cell line compared with normal liver cell line LO2 (Figure 3F). While the expression level of SPAG4, and MMRN1 were no significant between cell lines HepG2 and LO2 (Figure 3F).

### Establishment and validation of the HPRS model with good performance

**Figure 4:**
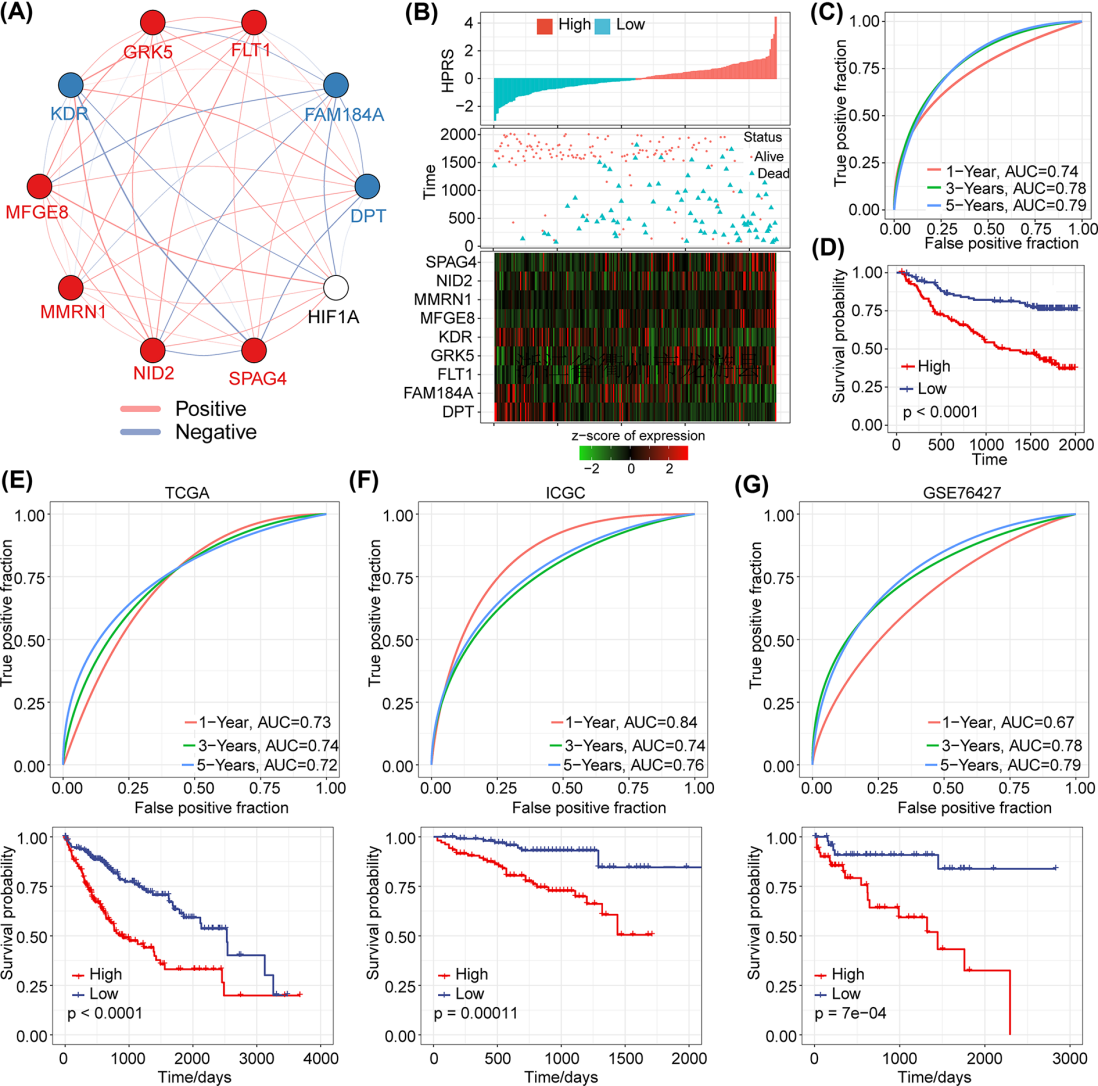
Establishment and validation of the HPRS risk model (**A**) The correlations of the candidate gene with HIF1A expression. (**B**) Risk score (top), patient status (middle), and mRNA expression heatmap (bottom) of the HPRS model for the GSE14520 training set. (**C**) ROC curve of the HPRS model in the training set. (**D**) The different risk groups had significant differences in overall survival time. (**E**–**G**) Time-dependent ROC and Kaplan–Meier curves of the HPRS model for the TCGA, ICGC, and GSE76427 cohorts.

Among the nine hypoxia-related genes previously identified, we found that six risk genes were positively correlated with HIF1A, and the other three protective genes were negatively correlated with HIF1A (Figure 4A). Then, we calculated the HPRS for each sample and normalized it. The HPRS distribution of the patients in the GSE14520 training dataset is shown in Figure 4B, which suggests that high-HPRS samples have a worse prognosis. Among the genes, low expression of DPT, FAM184A, and KDR was related to high risk, so these genes were considered protective factors, while high expression of FLT1, GRK5, MFGE8, MMRN1, NID2, and SPAG4 was associated with high risk, so these genes were considered risk factors. Furthermore, we used the R software package ‘timeROC’ to perform receiver-operating characteristic (ROC) analysis for the classification of prognosis based on HPRS. The areas under the ROC curve (AUCs) of the median HPRS value were 0.74, 0.78, and 0.79 for 1-, 3- and 5-year overall survival, respectively (Figure 4C). Patients in the low-HPRS group had a longer overall survival time than those in the high-HPRS group in the GSE14520 training cohort (Figure 4D). To confirm the robustness of the HPRS model, we used the same method to calculate the HPRS scores of the patients in the other three validation cohorts (TCGA, ICGC, and GSE76427). We observed similar results as those in the training set. High-HPRS patients have a worse prognosis, while low-HPRS patients have a better prognosis (Figure 4E–G).

### Enrichment analysis of the high- and low-HPRS groups

To further explore the difference in biological pathways between the high- and low-HPRS groups, we performed GSEA using all candidate gene sets in the Hallmark database [21], and FDR <0.05 was defined as significant enrichment. We found that many pathways (such as mTORC1 signaling, MYC target v1, MYC target v2, E2F targets, G2M checkpoint, mitotic spindle, and Wnt-β-catenin signaling) were activated in the high-HPRS group compared with the low-HPRS group in the GSE14520, TCGA, ICGC, and GSE76427 cohorts. In addition, the hypoxia pathway was activated in the high-HPRS group in the GSE14520 and TCGA cohorts (Supplementary Figure S1).

### Mutation characteristics of different HPRS groups

We further explored the differences in genomic changes between the high- and low-HPRS groups in the TCGA cohort. Compared with the low-HPRS group, the high-HPRS group showed a higher aneuploidy score, number of homologous recombination defects, fraction altered, number of segments, and tumor mutation burden (Supplementary Figure S2A). Additionally, we conducted a correlation analysis between HPRS and aneuploidy score, homologous recombination defects, fraction altered, number of segments, and tumor mutation burden, and we found that HPRS was significantly positively correlated with these factors (except for tumor mutation burden) (Supplementary Figure S2B). Furthermore, we found that the mutation frequencies of the TP53, ATAD2, ARHGEF10L, and ANK1 genes were higher in the high-HPRS group than in the low-HPRS group. For copy number variations, the high-HPRS group had a higher number of copy number amplifications and deletions than the low-HPRS group (Supplementary Figure S2C). Overall, the landscape of genetic variations in different HPRS groups might provide clues for explaining the different prognoses of HCC patients.

### Immune cell infiltration characteristics of the TME of different HPRS groups

**Figure 5:**
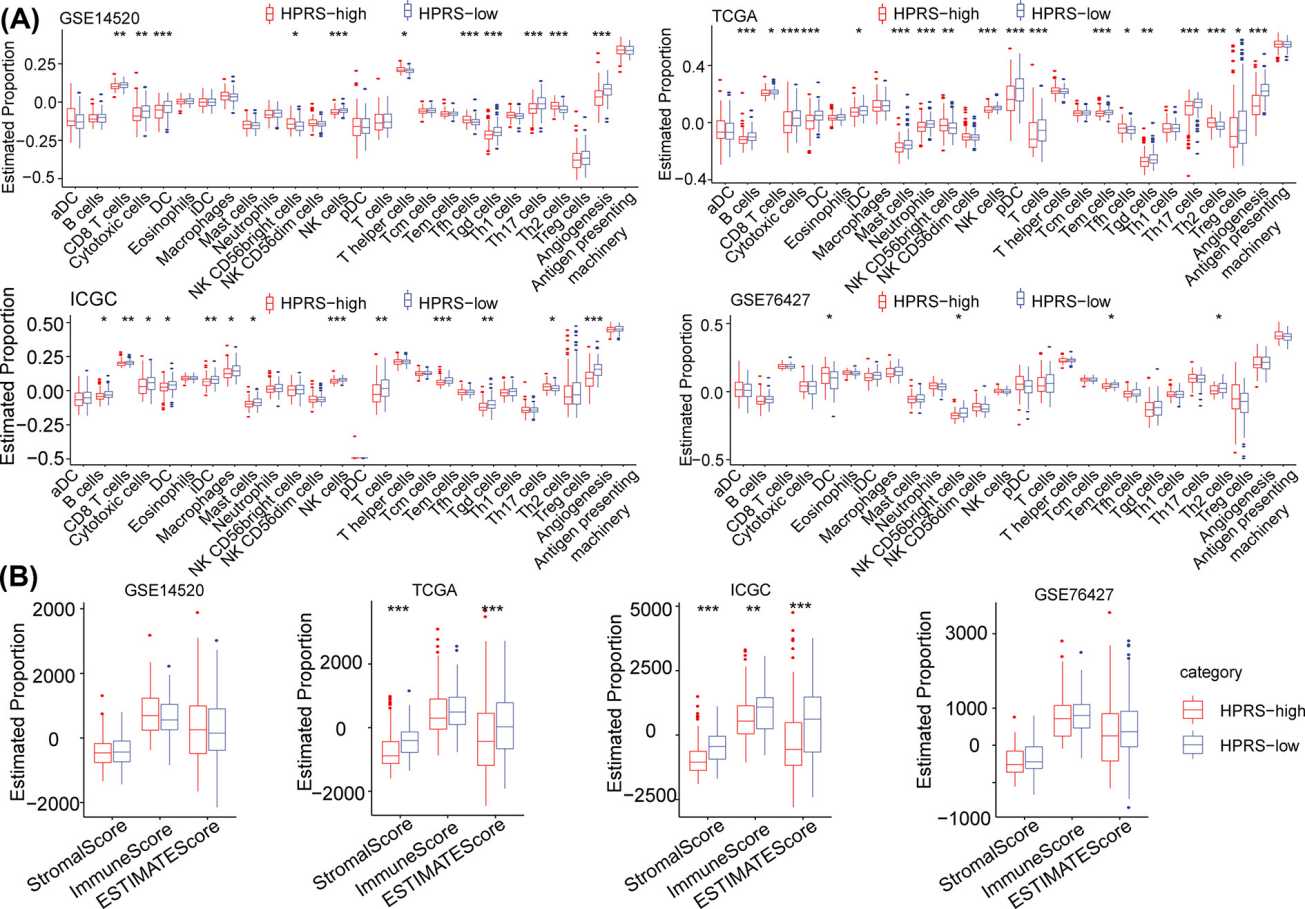
Immune cell infiltration characteristics of the TME of different HPRS groups (**A**) Expression levels of gene markers in immune cells were utilized to assess the immune cell infiltration features of HPRS subgroups in the four cohorts (GSE14520, TCGA, ICGC, and GSE76427 cohorts). (**B**) Immune cell infiltration was evaluated using the ESTIMATE method.

To further clarify the association between HPRS and infiltrating immune cells in the TME, we used the expression levels of gene markers on immune cells to assess the degree of immune cell infiltration of patients in the four cohorts (GSE14520, TCGA, ICGC, and GSE76427 cohorts) [24]. We found that various immune cells, such as CD8 T cells, cytotoxic cells, and dendritic cells, were significantly enriched in the low-HPRS group compared with the high-HPRS group (except GSE76427) (Figure 5A). We also used ESTIMATE to evaluate immune cell infiltration, and the results showed that the low-HPRS group in the TCGA, ICGC, and GSE76427 cohorts had a higher ImmuneScore than the high-HPRS group, which had relatively high immune cell infiltration (Figure 5B).

### Assessment of the predictive efficacy of the HPRS model for immunotherapy

**Figure 6:**
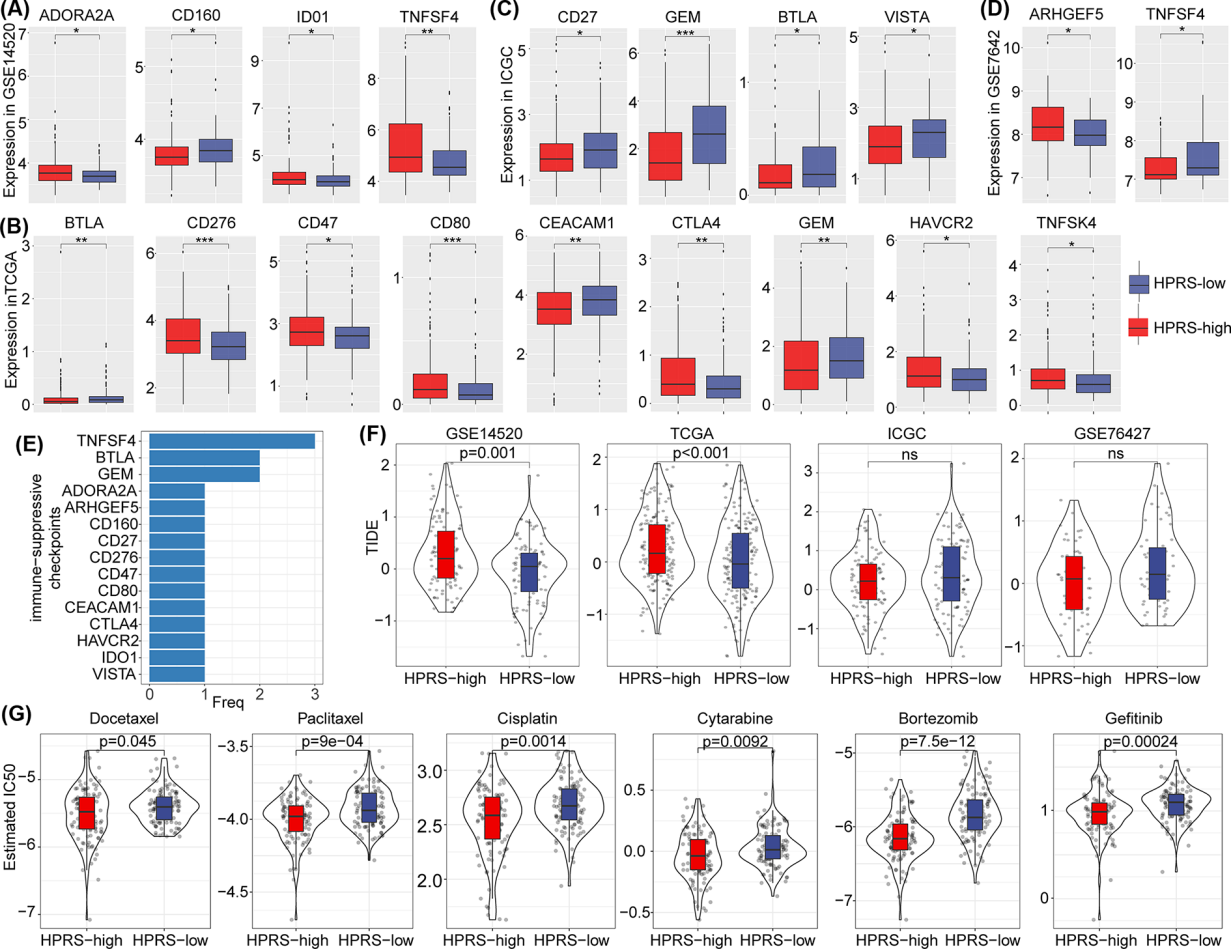
Assessment of the predictive efficacy of the HPRS model for immunotherapy (**A**–**D**) The difference in immune-suppressive checkpoints between the high- and low-HPRS groups in the four cohorts (GSE14520, TCGA, ICGC, and GSE76427 cohorts). (**E**) The TNFSF4, BTLA, and GEM immune-suppressive checkpoints were significantly different in the HPRS subgroups. (**F**) The prediction effect of the HPRS model was assessed using TIDE. (**G**) Different drug responses to traditional chemotherapy drugs (docetaxel, paclitaxel, cisplatin, cytarabine, bortezomib, and gefitinib) between the different HPRS groups in the GSE14520 cohort.

We next evaluated whether the HPRS model could serve as a biomarker for immunotherapy response. Here, all immune-suppressive checkpoints were obtained from the HisgAtlas database [26]. Four immune-suppressive checkpoints, ADORA2A, CD160, IDO1, and TNFSF4, were found to be significantly different between the high- and low-HPRS groups in the GSE14520 cohort (Figure 6A). A total of nine immune-suppressive checkpoints (BTLA, C276, CD47, CD80, CEACAM1, CTLA4, GEM, HAVCR2, and TNFSF4) were identified as significantly different in the HPRS groups in the TCGA cohort (Figure 6B). The immune-suppressive checkpoints CD27, GEM, BTLA, VISTA, ARHGEF5, and TNFSF4 were significantly different between the high- and low-HPRS groups in the ICGC and GSE76427 cohorts (Figure 6C,D). Based on the expression of immune-suppressive checkpoints in the four cohorts, we found that the TNFSF4, BTLA, and GEM immune checkpoints were significantly different between the high- and low-HPRS groups (Figure 6E). Additionally, we evaluated the predictive effect of the HPRS model using TIDE (http://tide.dfci.harvard.edu/) software. The higher the TIDE prediction score is, the higher the possibility of immune escape, indicating that the patient is less likely to benefit from immunotherapy (Figure 6F). We observed that the TIDE score was higher in the high-HPRS group than in the low-HPRS group in the GSE14520 and TCGA cohorts, suggesting that patients in the high-HPRS group have a higher possibility of immune escape and are less likely to benefit from immunotherapy. We also analyzed the different drug responses to traditional chemotherapy drugs (docetaxel, paclitaxel, cisplatin, cytarabine, bortezomib, and gefitinib) between the different HPRS groups in the GSE14520 cohort, and the results showed that patients in the low-HPRS group were more sensitive to these six chemotherapeutics (Figure 6G). Overall, the HPRS model could potentially be applied to predict patient response to HCC immunotherapy and chemotherapy.

### The HPRS model has a better prognostic ability than traditional clinical features

**Figure 7:**
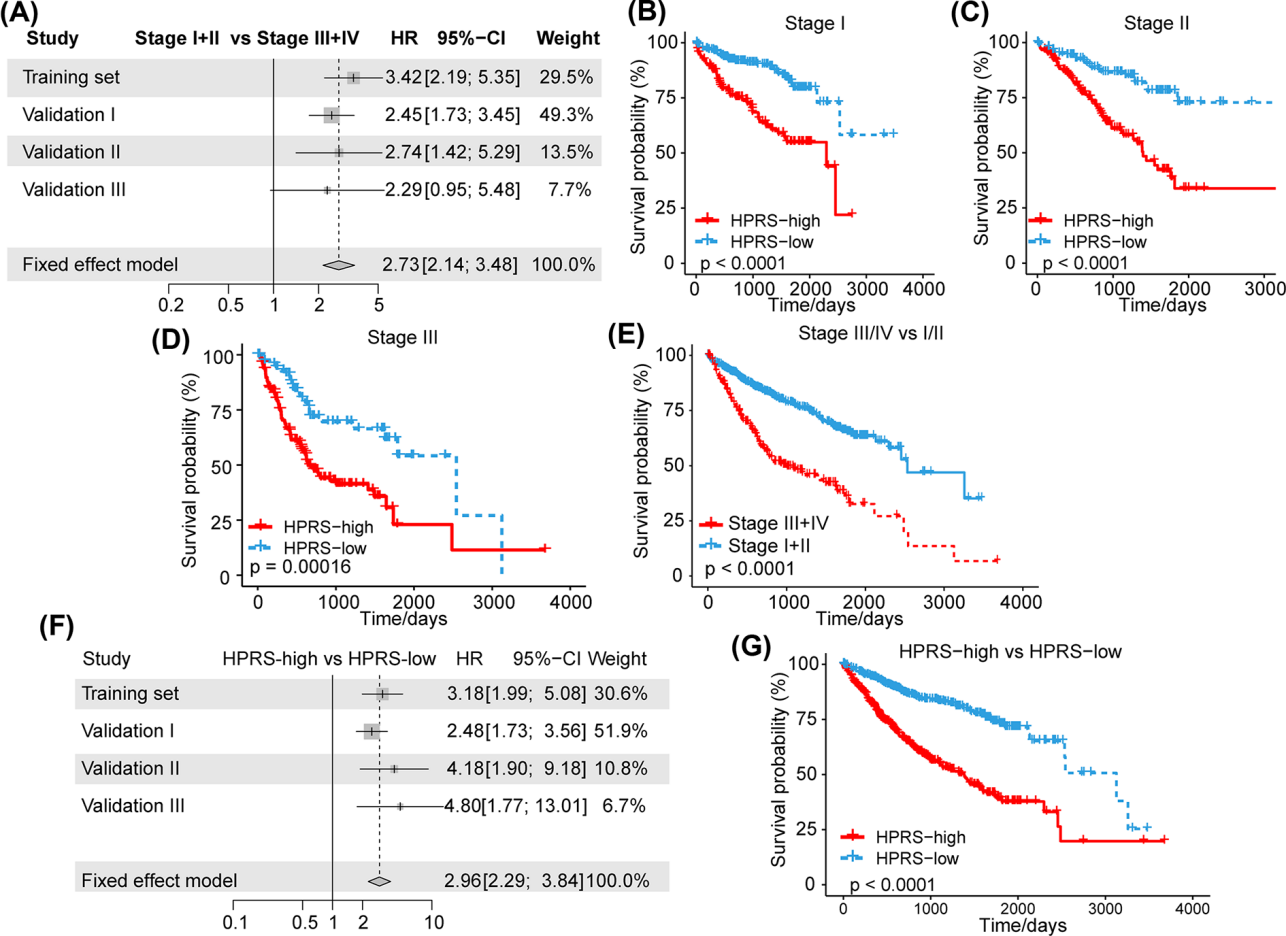
The HPRS model has a better prognostic ability than traditional clinical features (**A**) Meta-analysis showed that patients with a high TNM stage had a worse prognosis (HR = 2.73, 95% CI = 2.14–5.48). (**B**–**D**) Patients in stages I, II, and III with a high HPRS had a worse prognosis than those with a low HPRS (HR = 2.96, 95% CI = 2.29–3.84). (**E**) Survival difference of all patients in stages III+IV and I+II (**F**) Meta-analysis showed that among the 913 patients, those with a higher HPRS had a poorer prognosis than those with a lower HPRS (HR = 2.96, 95% CI = 2.29–3.84). (**G**) Patients in the high-HPRS group had a worse prognosis than those in the low-HPRS group among the 913 HCC patients.

To further explore the clinical application value of the HPRS model, we downloaded a total of 913 HCC samples by merging the training set and the three validation set datasets. The results of the meta-analysis showed that patients with a high TNM stage had a worse prognosis (hazard ratio (HR) = 2.73, 95% CI = 2.14–5.48) (Figure 7A). We also found that patients with stages I, II, and III disease with high HPRS had a worse prognosis than patients with low HPRS (HR = 2.96, 95% CI = 2.29–3.84) (Figure 7B–D). Concomitantly, we analyzed the survival difference of all patients in stages III+IV and I+II. The results showed that patients with stages I+II disease had a better prognosis than those with stages III+IV disease (Figure 7E). Furthermore, a meta-analysis demonstrated that among 913 patients, those with a higher HPRS had a poorer prognosis than those with a lower HPRS (HR = 2.96, 95% CI = 2.29–3.84) (Figure 7F). The survival analysis results showed that of the 913 HCC patients, those in the high-HPRS group had a significantly worse prognosis than those in the low-HPRS group (Figure 7G). The above results showed that HPRS might serve as a promising indicator for prognosis.

### HPRS combined with clinicopathological characteristics could further improve its prognostic prediction ability

**Figure 8:**
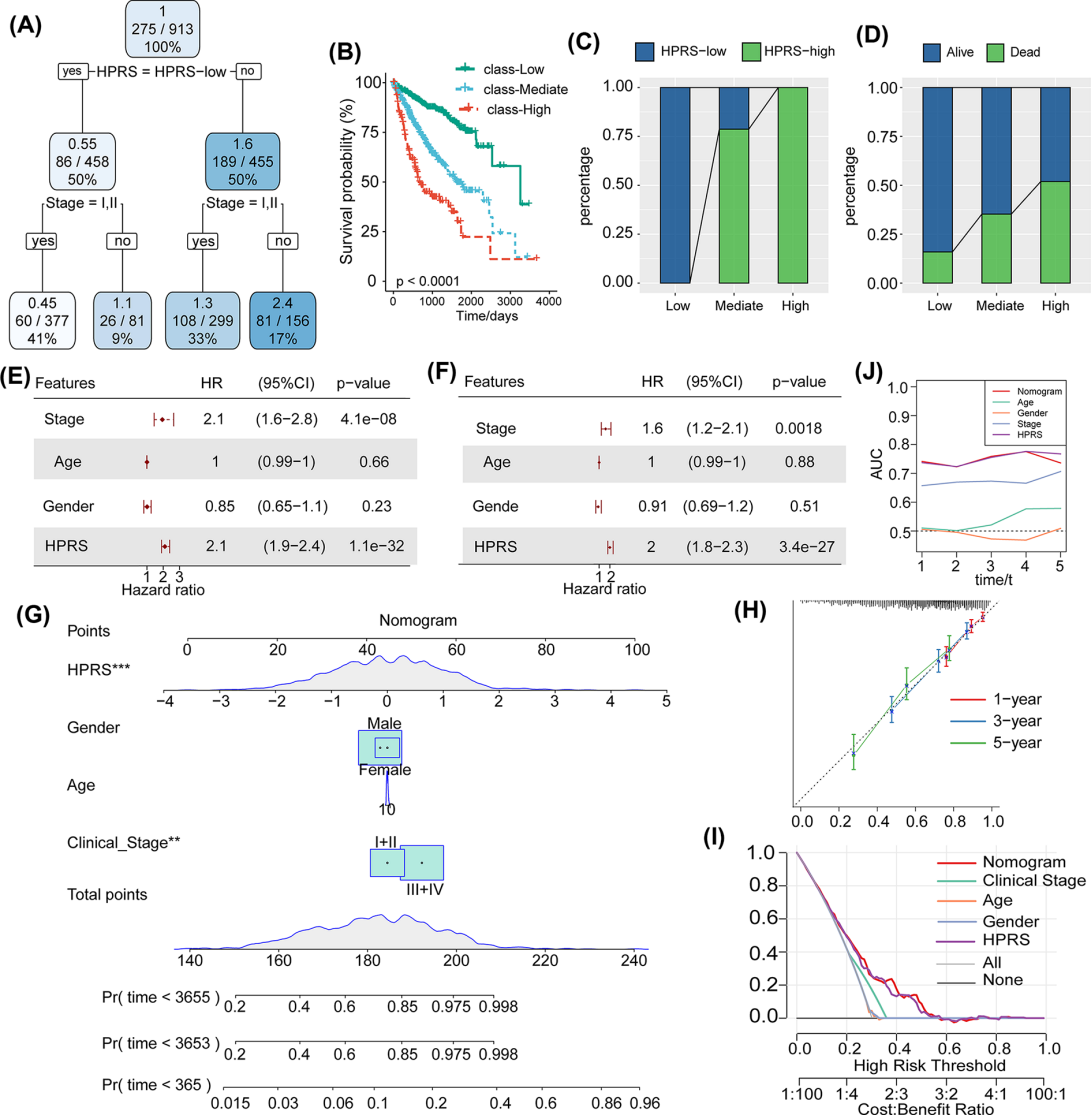
HPRS combined with clinicopathological characteristics could further improve its prognostic prediction ability (**A**) A survival decision tree was constructed based on four clinicopathological parameters of patients, including age, sex, TNM staging, and HPRS, in the whole dataset. (**B**) There were significant differences in overall survival among the three risk subgroups. (**C**) Patients in the high-risk group were all high-HPRS patients, and patients in the low-risk group were all low-HPRS patients. (**D**) A higher percentage of deaths was found in the high-risk group than in the medium-risk and low-risk groups. (**E,F**) Univariate and multivariate Cox regression analyses of HPRS and clinicopathological characteristics. (**G**) A nomogram was generated with HPRS and other clinicopathological characteristics. (**H**) A calibration curve was generated to evaluate the prediction accuracy of the HPRS model. (**I,J**) DCA was performed to evaluate the reliability of the model and other clinicopathological characteristics.

To optimize risk stratification, we constructed a survival decision tree based on the four clinicopathological parameters of patient age, sex, TNM staging, and HPRS in the whole dataset. The results showed that only TNM staging and HPRS were screened in the decision tree, and four different risk subgroups were identified (Figure 8A). Among them, HPRS was the most powerful parameter, followed by TNM staging. Here, we defined patients with low HPRS and early TNM stage as the low-risk group, while the medium-risk group was characterized by low HPRS and late TNM stage as well as high HPRS and early TNM stage, and the high-risk group was characterized by high HPRS and late stage. There were significant differences in overall survival among the three subgroups, as shown in Figure 8B. Among them, patients in the high-risk group were all high-HPRS patients, and patients in the low-risk group were all low-HPRS patients (Figure 8C). Moreover, a higher percentage of deaths was found in the high-risk group than in the medium-risk and low-risk groups (Figure 8D). Univariate and multivariate Cox regression analyses of HPRS and clinicopathological characteristics showed that HPRS was the most significant prognostic factor (Figure 8E,F). To quantify the risk assessment and survival probability of HCC, we generated a nomogram combining HPRS and clinicopathological characteristics (Figure 8G). In the calibration analysis, the prediction calibration curves of the three calibration points at 1, 3, and 5 years were close to the ideal performance (Figure 8H). Additionally, we used decision curve analysis (DCA) to evaluate the reliability of the model. The benefits of HPRS and the nomogram were significantly higher than those of other clinicopathological characteristics, suggesting that both the nomogram and HPRS showed the strongest predictive abilities (Figure 8I,J).

## Discussion

Understanding the molecular mechanism of tumor progression is a key step for targeted therapy in HCC [27]. Increasing evidence has demonstrated that hypoxia and hypoxia-related genes regulate tumor initiation and progression by altering chemoresistance in HCC [28,29]. A systematic analysis of the effects of hypoxia-related prognostic genes may identify novel therapeutic targets for therapy in HCC.

In the present study, we evaluated clinicopathological characteristics and adopted the ssGSEA score to assess hypoxia-related genes based on the relationship between the genes in the module and the ssGSEA score and prognosis of patients. Similar to our findings, hypoxia-related genes could be used to predict prognosis and therapeutic responses in lung adenocarcinoma [30,31]. Sun et al. [31] found that hypoxia could function as an independent risk factor for patients with lung adenocarcinoma, and their hypoxia-related model could serve as an effective tool to identify high-risk patients with lung adenocarcinoma at an early stage. Shi et al. [30] found that hypoxia was a risk factor for overall survival in patients, and their HPRS model could be used to predict the survival time in lung adenocarcinoma. In summary, hypoxia-related genes could be novel biomarkers for the survival prediction of patients with HCC.

We identified nine core hypoxia-related genes to construct a model for predicting the survival, clinicopathological characteristics, and immunotherapy response of HCC. Consistent with our findings, Zhang et al. found that immune-related lncRNA pairs could be used to construct a prognostic risk model in pancreatic cancer, and the three lncRNA pairs could be novel tumor treatment targets through the regulation of immune cells and tumor migration [32]. In a recent study, they developed a novel prognostic model based on the methylated and expressed genes in PD-1-negative patients with HCC, and the model had predictive power for 1-, 3-, and 5-year survival [33]. Therefore, further investigation of the roles of these core genes could provide clues for a deeper understanding of tumor progression.

We found that down-regulated DPT, FAM184A, and KDR and up-regulated FLT1, GRK5, MFGE8, MMRN1, NID2, and SPAG4 were risk factors for the prognosis of HCC patients. Similar to our study, a recent study showed that DPT expression was markedly decreased in HCC tissues, and decreased DPT expression was significantly correlated with a long survival time in patients with HCC. Mechanistically, overexpressed DPT inhibited cell proliferation *in vitro* and *in vivo* [34]. Furthermore, previous studies found that MFGE8 could promote tumor progression and might be a novel biomarker for the diagnosis of patients with HCC [35,36]. Interestingly, Liu et al. [37] found that the up-regulated CRKL-FLT1 pair was significantly associated with poor prognosis in patients with HCC, and knockdown of FLT1 inhibited HCC cell migration *in vitro*. Thus, further detection of the nine core genes may help us better understand the mechanism of HCC progression.

## Conclusion

We constructed a prognostic HPRS model based on hypoxia-related genes, which has high prognostic prediction accuracy in multiple independent datasets. Importantly, HPRS also had stable predictive performance for immunotherapy and chemotherapy of HCC.

## Supplementary Material

Supplementary Figures S1-S2 and Table S1

## Data Availability

All data were presented in the article.

## Abbreviations

ALT: alanine aminotransferase
AUC: areas under the ROC curve
CI: confidence interval
DC: dendritic cell
DCA: decision curve analysis
FDR: false discovery rate
GS: gene significance
GSEA: gene set enrichment analysis
HCC: hepatocellular carcinoma
HPRS: hypoxia-related prognostic risk score
HR: hazard ratio
ICGC: International Cancer Genome Consortium
LASSO: least absolute shrinkage and selection operator
LIHC: liver hepatocellular carcinoma
MM: module membership
ROC: receiver operating characteristic
ssGSEA: single-sample gene set enrichment analysis
TCGA: The Cancer Genome Atlas
TNM: tumor-node-metastasis
WGCNA: weighted gene coexpression network analysis

## References

[1] Bray F., Ferlay J., Soerjomataram I., Siegel R.L., Torre L.A. and Jemal A. (2018) Global cancer statistics 2018: GLOBOCAN estimates of incidence and mortality worldwide for 36 cancers in 185 countries. CA Cancer J. Clin. 68, 394–424 10.3322/caac.21492 30207593

[2] Villanueva A. (2019) Hepatocellular carcinoma. N. Engl. J. Med. 380, 1450–1462 10.1056/NEJMra1713263 30970190

[3] Nault J.C. and Villanueva A. (2021) Biomarkers for hepatobiliary cancers. Hepatology 73, 115–127 10.1002/hep.31175 32045030

[4] Shao C., Yang F., Miao S., Liu W., Wang C., Shu Y. et al. (2018) Role of hypoxia-induced exosomes in tumor biology. Mol. Cancer 17, 120 10.1186/s12943-018-0869-y 30098600 PMC6087002

[5] Jing X., Yang F., Shao C., Wei K., Xie M., Shen H. et al. (2019) Role of hypoxia in cancer therapy by regulating the tumor microenvironment. Mol. Cancer 18, 157 10.1186/s12943-019-1089-9 31711497 PMC6844052

[6] Wu X.Z., Xie G.R. and Chen D. (2007) Hypoxia and hepatocellular carcinoma: the therapeutic target for hepatocellular carcinoma. J. Gastroenterol. Hepatol. 22, 1178–1182 10.1111/j.1440-1746.2007.04997.x 17559361

[7] Graham K. and Unger E. (2018) Overcoming tumor hypoxia as a barrier to radiotherapy, chemotherapy and immunotherapy in cancer treatment. Int. J. Nanomedicine 13, 6049–6058 10.2147/IJN.S140462 30323592 PMC6177375

[8] Erler J.T. and Giaccia A.J. (2006) Lysyl oxidase mediates hypoxic control of metastasis. Cancer Res. 66, 10238–10241 10.1158/0008-5472.CAN-06-3197 17079439

[9] Xiong X.X., Qiu X.Y., Hu D.X. and Chen X.Q. (2017) Advances in hypoxia-mediated mechanisms in hepatocellular carcinoma. Mol. Pharmacol. 92, 246–255 10.1124/mol.116.107706 28242743

[10] Kyrochristos I.D., Ziogas D.E. and Roukos D.H. (2019) Dynamic genome and transcriptional network-based biomarkers and drugs: precision in breast cancer therapy. Med. Res. Rev. 39, 1205–1227 10.1002/med.21549 30417574

[11] Yin F., Shu L., Liu X., Li T., Peng T., Nan Y. et al. (2016) Microarray-based identification of genes associated with cancer progression and prognosis in hepatocellular carcinoma. J. Exp. Clin. Cancer Res. 35, 127 10.1186/s13046-016-0403-2 27567667 PMC5002170

[12] Zhang B., Tang B., Gao J., Li J., Kong L. and Qin L. (2020) A hypoxia-related signature for clinically predicting diagnosis, prognosis and immune microenvironment of hepatocellular carcinoma patients. J. Transl. Med. 18, 342 10.1186/s12967-020-02492-9 32887635 PMC7487492

[13] Hu B., Yang X.B. and Sang X.T. (2020) Development and verification of the hypoxia-related and immune-associated prognosis signature for hepatocellular carcinoma. J. Hepatocell. Carcinoma 7, 315–330 10.2147/JHC.S272109 33204664 PMC7667586

[14] Wang M., Wang L., Pu L., Li K., Feng T., Zheng P. et al. (2020) LncRNAs related key pathways and genes in ischemic stroke by weighted gene co-expression network analysis (WGCNA). Genomics 112, 2302–2308 10.1016/j.ygeno.2020.01.001 31923616

[15] Hou J., Ye X., Li C. and Wang Y. (2021) K-Module algorithm: an additional step to improve the clustering results of WGCNA co-expression networks. Genes (Basel) 12, 87 10.3390/genes12010087 33445666 PMC7828115

[16] Gao J., Kwan P.W. and Shi D. (2010) Sparse kernel learning with LASSO and Bayesian inference algorithm. Neural Netw. 23, 257–264 10.1016/j.neunet.2009.07.001 19604671

[17] McEligot A.J., Poynor V., Sharma R. and Panangadan A. (2020) Logistic LASSO regression for dietary intakes and breast cancer. Nutrients 12, 2652 10.3390/nu12092652 32878103 PMC7551912

[18] Oprescu S.N., Horzmann K.A., Yue F., Freeman J.L. and Kuang S. (2018) Microarray, IPA and GSEA analysis in mice models. Bio. Protoc. 8, e2999 10.21769/BioProtoc.2999 30547052 PMC6289195

[19] Canzler S. and Hackermüller J. (2020) multiGSEA: a GSEA-based pathway enrichment analysis for multi-omics data. BMC Bioinformatics 21, 561 10.1186/s12859-020-03910-x 33287694 PMC7720482

[20] Subramanian A., Tamayo P., Mootha V.K., Mukherjee S., Ebert B.L., Gillette M.A. et al. (2005) Gene set enrichment analysis: a knowledge-based approach for interpreting genome-wide expression profiles. Proc. Natl. Acad. Sci. U.S.A. 102, 15545–15550 10.1073/pnas.0506580102 16199517 PMC1239896

[21] Liberzon A., Birger C., Thorvaldsdóttir H., Ghandi M., Mesirov J.P. and Tamayo P. (2015) The Molecular Signatures Database (MSigDB) hallmark gene set collection. Cell Syst. 1, 417–425 10.1016/j.cels.2015.12.004 26771021 PMC4707969

[22] Barbie D.A., Tamayo P., Boehm J.S., Kim S.Y., Moody S.E., Dunn I.F. et al. (2009) Systematic RNA interference reveals that oncogenic KRAS-driven cancers require TBK1. Nature 462, 108–112 10.1038/nature08460 19847166 PMC2783335

[23] Xiao B., Liu L., Li A., Xiang C., Wang P., Li H. et al. (2020) Identification and verification of immune-related gene prognostic signature based on ssGSEA for osteosarcoma. Front. Oncol. 10, 607622 10.3389/fonc.2020.607622 33384961 PMC7771722

[24] Şenbabaoğlu Y., Gejman R.S., Winer A.G., Liu M., Van Allen E.M., de Velasco G. et al. (2016) Tumor immune microenvironment characterization in clear cell renal cell carcinoma identifies prognostic and immunotherapeutically relevant messenger RNA signatures. Genome Biol. 17, 231 10.1186/s13059-016-1092-z 27855702 PMC5114739

[25] Colaprico A., Silva T.C., Olsen C., Garofano L., Cava C., Garolini D. et al. (2016) TCGAbiolinks: an R/Bioconductor package for integrative analysis of TCGA data. Nucleic. Acids. Res. 44, e71 10.1093/nar/gkv1507 26704973 PMC4856967

[26] Liu Y., He M., Wang D., Diao L., Liu J., Tang L. et al. (2017) HisgAtlas 1.0: a human immunosuppression gene database. Database (Oxford) 2017, bax094 10.1093/database/bax094 31725860 PMC7243927

[27] He Q., Liu M., Huang W., Chen X., Zhang B., Zhang T. et al. (2021) IL-1β-induced elevation of solute carrier family 7 member 11 promotes hepatocellular carcinoma metastasis through up-regulating programmed death ligand 1 and colony-stimulating factor 1. Hepatology, 3174–3193 10.1002/hep.32062 34288020

[28] Gao R., Buechel D., Kalathur R.K.R., Morini M.F., Coto-Llerena M., Ercan C. et al. (2021) USP29-mediated HIF1α stabilization is associated with Sorafenib resistance of hepatocellular carcinoma cells by upregulating glycolysis. Oncogenesis 10, 52 10.1038/s41389-021-00338-7 34272356 PMC8285469

[29] Zeng Z., Lu Q., Liu Y., Zhao J., Zhang Q., Hu L. et al. (2021) Effect of the hypoxia inducible factor on sorafenib resistance of hepatocellular carcinoma. Front Oncol. 11, 641522 10.3389/fonc.2021.641522 34307125 PMC8292964

[30] Shi R., Bao X., Unger K., Sun J., Lu S., Manapov F. et al. (2021) Identification and validation of hypoxia-derived gene signatures to predict clinical outcomes and therapeutic responses in stage I lung adenocarcinoma patients. Theranostics 11, 5061–5076 10.7150/thno.56202 33754044 PMC7978303

[31] Sun J., Zhao T., Zhao D., Qi X., Bao X., Shi R. et al. (2020) Development and validation of a hypoxia-related gene signature to predict overall survival in early-stage lung adenocarcinoma patients. Ther. Adv. Med. Oncol. 12, 1758835920937904 10.1177/1758835920937904 32655701 PMC7333486

[32] Zhang Q., Wang Z., Yu X., Zhang M., Zheng Q., He Y. et al. (2021) Immune subtypes based on immune-related lncRNA: differential prognostic mechanism of pancreatic cancer. Front Cell Dev. Biol. 9, 698296 10.3389/fcell.2021.698296 34307375 PMC8292792

[33] Zhu L. and Guo W. (2021) Combined DNA methylation and transcriptomic assessments to determine a prognostic model for PD-1-negative hepatocellular carcinoma. Front Cell Dev. Biol. 9, 708819 10.3389/fcell.2021.708819 34458266 PMC8385720

[34] Liu S., Qiu J., He G., Geng C., He W., Liu C. et al. (2020) Dermatopontin inhibits WNT signaling pathway via CXXC finger protein 4 in hepatocellular carcinoma. J. Cancer 11, 6288–6298 10.7150/jca.47157 33033513 PMC7532498

[35] Ko D.S., Kim S.H., Park J.Y., Lee G., Kim H.J., Kim G. et al. (2020) Milk fat globule-EGF factor 8 contributes to progression of hepatocellular carcinoma. Cancers (Basel) 12, 403 10.3390/cancers12020403 PMC7072366 32050643

[36] Shimagaki T., Yoshio S., Kawai H., Sakamoto Y., Doi H., Matsuda M. et al. (2019) Serum milk fat globule-EGF factor 8 (MFG-E8) as a diagnostic and prognostic biomarker in patients with hepatocellular carcinoma. Sci. Rep. 9, 15788 10.1038/s41598-019-52356-6 31673081 PMC6823494

[37] Liu C.H., Chen T.C., Chau G.Y., Jan Y.H., Chen C.H., Hsu C.N. et al. (2013) Analysis of protein-protein interactions in cross-talk pathways reveals CRKL protein as a novel prognostic marker in hepatocellular carcinoma. Mol. Cell. Proteomics 12, 1335–1349 10.1074/mcp.O112.020404 23397142 PMC3650343

