# Supplementary material for: A hypoxia-related prognostic model predicts overall survival and treatment response in hepatocellular carcinoma
Source: Biosci Rep. 2022 Nov 18;42(11):BSR20221089. doi: 10.1042/BSR20221089 (PMC9679399; doi:10.1042/BSR20221089)
Supplement: Supplementary Figures S1-S2 and Table S1 [file BSR-2022-1089_supp.pdf]

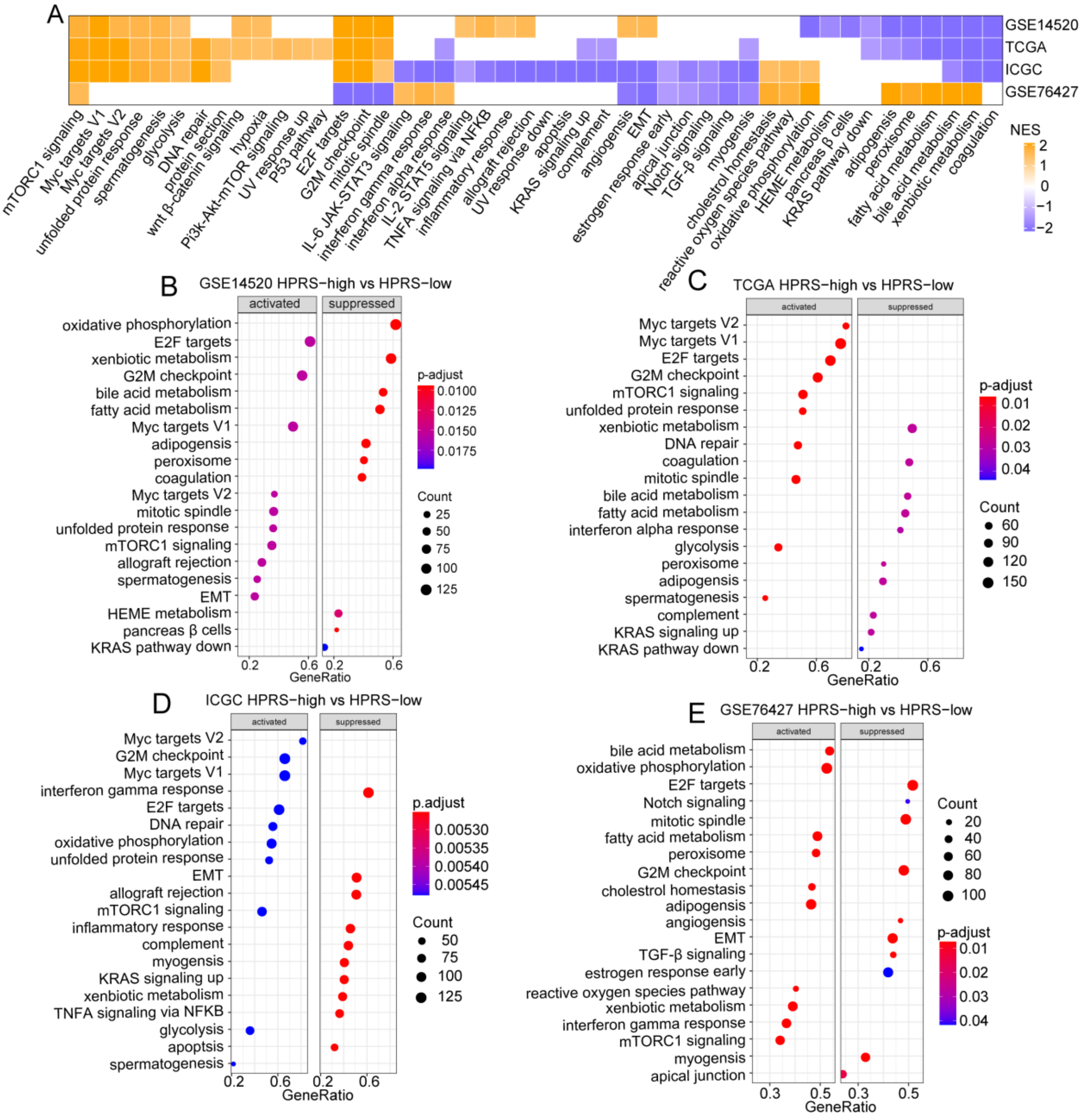

FIGURE S1 | Enrichment analysis of the high- and low-HPRS groups.

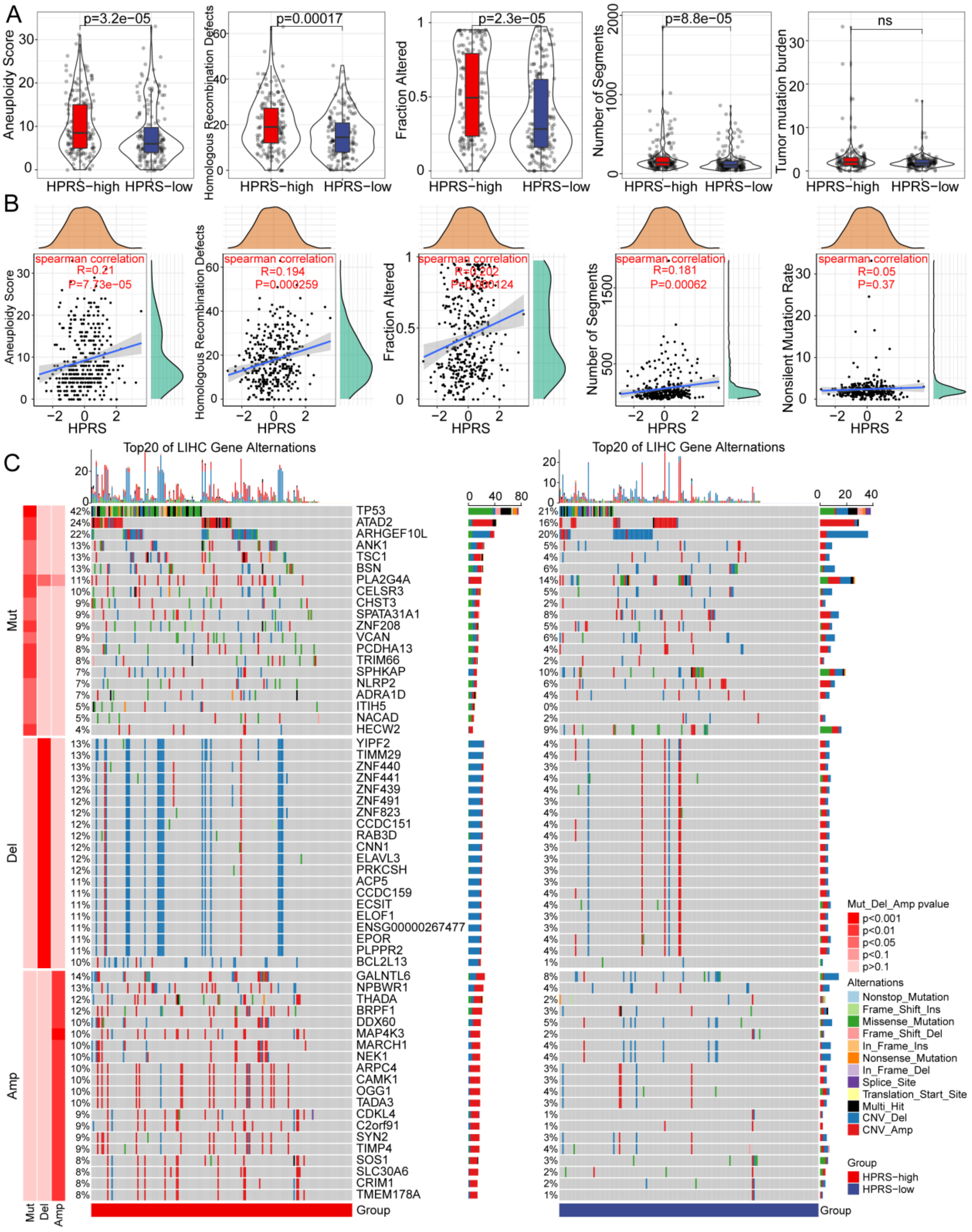

FIGURE S2 | Mutation characteristics of the different HPRS groups. **(A)** The differences in genomic changes between the high- and low-HPRS groups in the TCGA cohort. **(B)** Correlation analysis between HPRS and genomic changes. **(C)** Correlation analysis of different HPRS groups and gene mutation frequency and copy number variation.

Table S1. The information of primers sequences for qRT-PCR assay.

Primer name	Sequence (5'-3')
GAPDH-F	CAAGGCTGTGGGCAAGGTCATC
GAPDH-R	GTGTCGCTGTTGAAGTCAGAGGAG
FLT1- F	CCAAATAAGCACACCACGCC
FLT1-R	TGCTTTGGTCAATTCGTCGC
DPT-F	CGCTACTTCGAGTCAGTGCT
DPT-R	AAGTGGTTGTTGCTCCTCGG
KDR-F	TCTCTGCCTACCTCACCTGTTTCC
KDR-R	CACTGTCCGTCTGGTTGTCATCTG
FAM184A-F	TGCAGCAAGAGATTCATGGC
FAM184A-R	GTAAGCCGCTGTCGTTCTTG
SPAG4-F	TCTCCAGTAGTCTCTGAGGAGC
SPAG4-R	CGGATGGAACAGACCTCCC
MMRN1-F	GGCATTGGGCTTAACAACAGT
MMRN1-R	CGACATGACCCGAGTGGTT
MFGE8-F	CCTGCCACAACGGTGGTTTAT
MFGE8-R	CACATTTCGTCTCACAGTGGTT
GRK5-F	TGGGCTGGAGTGTTACATTCA
GRK5-R	GGGGTGAGGTACTTGGTCATAAT
NID2-F	GCCATAGACCACATCCGCAGAAC
NID2-R	CCCAGCAGAGCAGTTTAGAGAAAGG
